# Is the β_3_-Adrenoceptor a Valid Target for the Treatment of Obesity and/or Type 2 Diabetes?

**DOI:** 10.3390/biom13121714

**Published:** 2023-11-28

**Authors:** Haneen S. Dwaib, Martin C. Michel

**Affiliations:** 1 Department of Clinical Nutrition and Dietetics, Palestine Ahliya University, Bethlehem P.O. Box 1041, Palestine; haneen.dwaib@paluniv.edu.ps; 2 Department of Pharmacology, University Medical Center, Johannes Gutenberg University, Langenbeckstr. 1, 55131 Mainz, Germany

**Keywords:** β_3_-adrenoceptor, obesity, type 2 diabetes, species difference, insulin release, glucose uptake, thermogenesis

## Abstract

β_3_-Adrenoceptors mediate several functions in rodents that could be beneficial for the treatment of obesity and type 2 diabetes. This includes promotion of insulin release from the pancreas, cellular glucose uptake, lipolysis, and thermogenesis in brown adipose tissue. In combination, they lead to a reduction of body weight in several rodent models including ob/ob mice and Zucker diabetic fatty rats. These findings stimulated drug development programs in various pharmaceutical companies, and at least nine β_3_-adrenoceptor agonists have been tested in clinical trials. However, all of these projects were discontinued due to the lack of clinically relevant changes in body weight. Following a concise historical account of discoveries leading to such drug development programs we discuss species differences that explain why β_3_-adrenoceptors are not a meaningful drug target for the treatment of obesity and type 2 diabetes in humans.

## 1. Introduction

Obesity is generally defined as a body mass index exceeding 30 kg/m^2^. Its prevalence has markedly increased in past decades, irrespective of ethnicity, gender, socioeconomic status, or geographical location. Based on affecting one third of the global population, it is now considered to be a pandemic [1]. Obesity increases the risk for multiple conditions, most importantly type 2 diabetes (T2DM). Other conditions occurring more frequently in obese subjects include lower urinary symptoms such as the overactive bladder syndrome [2], which is the only established and approved indication for β_3_-adrenoceptor (AR) agonists [3].

Behavioral modification is the first line management of obesity but often is insufficiently effective, leading to a need for medical or even surgical treatment. Agonists of glucagon-like peptide 1 receptors such as semaglutide have recently proven to be effective pharmacological treatments of obesity, but not all obese subjects are responsive to this drug class and, even in responders, their use can be limited by tolerability issues [4]. Therefore, a continuing medical need exists for alternative pharmacological treatment options. This manuscript discusses how the idea of targeting β_3_-AR for the treatment of obesity and T2DM developed and which evidence supports and argues against the validity of this concept.

In principle, a reduction of body weight and/or improvement of hyperglycemia can be achieved by three main means. A reduction of caloric intake (dieting) is a domain of behavioral modification, but the recently introduced glucagon-like peptide 1 receptor agonists also use this mechanism [4,5]. However, there is no evidence linking β_3_-AR to control of food intake. A second approach is to increase energy expenditure leading to a negative energy balance if caloric intake does not increase to a similar extent. This can be attained by exercise (not covered here) and increased lipolysis and thermogenesis. More recently, an enhanced renal excretion of glucose has emerged as an additional mechanism to increase energy expenditure; this mechanism is used by sodium glucose transporter 2 inhibitors [6]. Various members of the latter drug class, including canagliflozin, dapagliflozin, and empagliflozin, have been found to decrease body weight in T2DM patients; however, with an average weight loss of <2 kg, these effects are too limited to be considered as anti-obesity drugs [7]. While limited evidence in rodents points to a role of β_3_-AR in the regulation of renal function [8], there is no evidence linking them to renal glucose excretion. A third option linked to glycemic control but not necessarily to body weight is modulation of insulin release from pancreatic β cells and/or cellular glucose uptake.

Brown adipose tissue (BAT) activation has been considered a corner stone in improving metabolic health because it is a metabolic sink for glucose and free fatty acids (FAA) and correlates to improved glucose and insulin sensitivity [9,10,11,12]. Therefore, it was suggested to target BAT by β_3_-AR agonists such as mirabegron as a potential treatment for metabolic dysfunction and obesity [12,13,14].

Following a concise review of historical aspects leading to the initiation of research and development programs in the β_3_-AR field, this manuscript will summarize limited data on modulation of insulin release and/or cellular glucose uptake and then mainly focus on their effects on lipolysis and thermogenesis. In line with their proposed mechanisms of action in T2DM, β_3_-AR agonists did not improve hyperglycemia in rodent models of type 1 diabetes (T1DM), e.g., as induced by streptozotocin or alloxan [15]; therefore, data related to T1DM will not be covered here systematically.

## 2. Historical Aspects

Soon after Lands had proposed a subdivision of β-AR into the subtypes β_1_ and β_2_ [16], emerging evidence supported that some responses to β-adrenergic agonists such as isoprenaline were not mediated by either of these two subtypes. Notably, this included evidence that an atypical receptor subtype, i.e., different from β_1_- and β_2_-AR, may mediate lipolysis in rodents [17,18,19]. Other physiological responses attributed to an atypical β-AR included inhibition of intestinal motility in rats [17,20] and relaxation of human urinary bladder [21]. The latter has meanwhile led to β_3_-AR agonists being a guideline recommended drug class for the treatment of the overactive bladder syndrome [3].

The initial research and development programs on β_3_-AR agonists as potential treatments of obesity and T2DM were stimulated by lipolysis and other functional studies [22]. The field gained traction upon the cloning of the human β_3_-AR [23] and its homologs of rats [24] and mice [25]. Of note, the latter study found that the rank order of potency of agonists to stimulate cAMP formation in transfected CHO cells correlated well with that to induce lipolysis in rat brown adipocytes. Moreover, the mRNA expression of the newly cloned rat receptor was reduced by 71% in obese Zucker rats as compared to lean animals. The interpretation of the findings from rodents was limited by the fact that the human and the rodent β_3_-AR genes differ in multiple ways. They differ in the presence and location of introns [26,27,28] and in their 5′ flanking regions and regulatory sequences [29], leading to possible differences in their transcriptional control.

### 2.1. Insight from Gene Polymorphism Association Studies

Soon after the cloning of the human β_3_-AR gene, it was reported that a Trp64Arg polymorphism in the coding region of the human β_3_-AR gene was associated with an increased capacity for weight gain [30] and an earlier onset of T2DM, particularly in Pima Indians [31]. While similar findings were obtained by some investigators [32,33,34,35], others did not confirm such associations, particularly in Caucasian populations [36,37,38]. Others reported an association of the polymorphism with some but not other parameters related to metabolism and obesity [39] or that the polymorphism was associated with responses to some but not other β_3_-AR agonists [33].

A major review of these and other studies, mostly on cardiovascular phenotypes, concluded that the available evidence was insufficient to establish a link between the Trp64Arg polymorphism and obesity or diabetes but indicated a trend for the Arg64 allele as being a possible risk factor [40]. This conclusion was also supported by lipolysis studies in adipocytes from genotyped obese and normal weight subjects upon stimulation with the atypical agonist CGP 12,177 [32] or similar studies involving subjects with multiple ethnicities and isoprenaline as the agonist [33,36]. Such a link would imply that the 64Arg variant of the receptor is hypofunctional. However, dedicated in vitro studies based on site-directed mutagenesis did not consistently find the 64Arg variant to be hypofunctional [41]. Importantly, the β_3_-AR locus has not shown up as a trait for obesity or diabetes in any of the genome-wide association studies. Evidence for an association of β_3_-AR gene polymorphisms and lower urinary tract function has also remained inconclusive [41].

### 2.2. Insight from β_3_-AR Knock-Out Studies

Independent groups have generated β_3_-AR knock-out mice that exhibited several metabolism related phenotypes. One set of knock-out mice exhibited a 34% and 131% increase in total body fat in males and females, respectively [42]. The other knock-out line, reporting on male mice only, had a 42% increase in total body fat [43], implying a sex difference in the role of β_3_-ARs in metabolic control. The latter line also had an attenuated respiratory rate response to the β_3_-AR agonist CL 316,243 in white adipose tissues (WAT) and BAT, whereas those to the β_1_- and β_2_-AR agonists dobutamine and terbutaline, respectively, were preserved [44]. Surprisingly at the time, the response to CGP 12,177 was attenuated in WAT but not BAT of the knock-out mice. This may be explained by more recent findings that CGP 12,177 is a partial agonist at β_3_-ARs, an orthosteric antagonist at the β_1_- and β_2_-AR, and an agonist at a non-orthosteric site of the β_1_-AR [45].

### 2.3. Early Research and Development Programs

A detailed account of the early research and development work on β_3_-AR agonists as potential treatments of obesity and T2DM has been provided [22]. A starting point had been observations that ephedrine [46,47,48] and other sympathomimetic agents including various β-AR agonists increased thermogenesis and caused weight loss in the genetically obese ob/ob mice [49,50].

While the initial studies suggested that the weight loss response involves a β-AR, the challenge became to find compounds that mimic this effect but do not cause hypertension, tachycardia, tremor, or hypokalemia, side effects known to be mediated by β_1_- and/or β_2_-ARs [51]. As some of these effects apparently exhibit non-linear receptor-effector coupling (receptor reserve, also known as spare receptors) [52], a high degree of selectivity for the β_3_-AR relative to the other subtypes is required to avoid such adverse effects [53].

The apparently first research program on β_3_-ARs as a drug target for the treatment of obesity and T2DM was launched at Beecham Pharmaceuticals (now part of GSK). It led to the discovery of atypical β-AR agonists including BRL 28,410, BRL 35,135 and BRL 37,344, which were effective on lipolysis but lacked the above side effects attributed to β_1_- and/or β_2_-ARs [54]. The compounds identified at Beecham Pharmaceuticals turned out to be agonists at the cloned β_3_-AR, although their selectivity for this subtype has meanwhile been challenged, particularly for the human β_3_-AR [55]. Subsequently, multiple companies filed patents disclosing β_3_-AR agonists and their potential use in the treatment of obesity and T2DM (see Section 8).

Taken together, the knock-out mouse studies supported a role for the β_3_-AR in the regulation of total body fat in rodents. In contrast, human gene polymorphism studies remained inconclusive, and the β_3_-AR gene locus did not point to a role of this receptor in obesity and diabetes in genome-wide association studies. With hindsight, these findings challenge the wisdom behind obesity and T2DM drug discovery programs targeting β_3_-AR.

## 3. Insulin Release and Cellular Glucose Uptake

A physiological function of insulin is stimulation of cellular glucose uptake mostly into adipocytes, e.g., after a meal. While this helps to acutely maintain euglycemia, it can chronically lead to excessive lipid storage, i.e., obesity. Therefore, several studies have addressed the effects of β_3_-AR agonists on insulin release and circulating insulin levels, and on the interaction of insulin and β_3_-ARs in the control of cellular glucose uptake.

### 3.1. Insulin Release

An early study reported that BRL 26,830 increased plasma insulin concentrations in fasted rats and improved glucose disposal after a glucose load in non-diabetic rats and mice [15]. Subsequent studies by others reported dose-dependent increases of blood insulin and glucagon concentrations along with a lowering of blood glucose in fasted mice; propranolol at 10–50 mg/kg attenuated these responses whereas metoprolol and ICI 118,551 at 50 mg/kg mimicked the propranolol response only partly [56]. No increase of blood insulin concentrations was observed in mice with streptozotocin-induced T1DM, implying that these effects required intact pancreatic β cells. Within the same report, BRL 26,830 also increased blood insulin and glucagon in dogs; while glucose levels were not affected, those of free fatty aicds (FFA) increased markedly, pointing to a site of action not in the pancreas but rather in adipose tissue (AT) in canines.

In cultured rat pancreatic islet cells, neither BRL 26,830 nor the related compound BRL 28,410 stimulated insulin release in the presence of 2.8 or 5.6 mM glucose. The same group also studied the β_3_-AR agonist CL 316,243 in in situ perfused mouse pancreas [57]. The agonist concentration-dependently stimulated insulin secretion, which was partially inhibited by propranolol and ICI 118,551, but not by metoprolol; based on an only incomplete inhibition by even high concentrations of propranolol (200 µM, expected to saturate β_1_- and β_2_-ARs), the investigators proposed that a major part of this effect was mediated by β_3_-ARs.

Experiments in the rat insulinoma cell line RIN 1040-38 (a model for pancreatic β cells) found β_3_-AR expression and increased insulin release in the presence of BRL 37,344 and CL 316,243 [58]. However, this release had two characteristics that complicate interpretation of the data. First, both agonists had bell-shaped concentration-response curves with maximal effects at 1–10 nM but a lack of effect at 100 nM. Second, the effect was transient with a peak after 30 min and return to control levels after 60 min. If the cells were transfected with the human wild-type β_3_-AR, the bell-shaped concentration-response curve remained; upon transfection with the 64Arg variant of the β_3_-AR, responses to CL 316,243 were not detected, again pointing to this variant being hypofunctional.

CL 316,243 increased pancreatic islet blood flow and plasma insulin concentration in rats while not affecting overall pancreatic blood flow [59]. This was prevented by a high dose of bupranolol (general β-AR antagonist also inhibiting β_3_-ARs) but not by nadolol (not inhibiting β_3_-ARs), implying involvement of a β_3_-AR. Based on these findings, the authors proposed that insulin release by a β_3_-AR agonist may occur at least partly secondary to vasodilation of microvessels in the islet of Langerhans. Of note, while vasodilation is typically attributed to β_2_-ARs, it can occur via β_3_-ARs in some vascular beds [60]. Others have proposed that the insulin release promoted by β_3_-AR agonists in mice may occur at least partly secondary to lipolysis and release of free fatty acids [61].

A study with three selective β_3_-AR agonists for 14 days in db/db mice, including solabegron that has been tested clinically in overactive bladder patients [62], reported dose-dependent reductions of plasma insulin concentrations [63], which may be secondary to overall metabolic improvements as shown by concomitant reductions of glucose and HbA1c.

The involvement of β_3_-AR in insulin release, food intake, and oxygen consumption induced by CL 316,243 in rat WAT was confirmed by experiments in genetically modified mouse models with either transgenic expression of the β_3_-AR in AT or in β_3_-AR knockout mice [64]. 

Taken together, these data indicate that β_3_-AR agonists can promote insulin release from the pancreas of rats and mice upon acute administration, but the underlying cellular and molecular mechanisms remain unclear and may be indirect, i.e., secondary to vasodilation. Moreover, these acute effects were not observed in dogs and, at least in hyperglycemic mice, turn into the opposite upon chronic administration.

These studies, in combination with the expression of β_3_-AR mRNA and protein in the human pancreas, specifically in the islets of Langerhans [58], have prompted limited investigations in human subjects. Eight healthy subjects received single oral doses of BRL 35,135 (8 mg) or salbutamol (8 mg) after pre-treatment with placebo, bisoprolol (5 mg) or nadolol (20 mg) [65]. Both agonists lowered serum potassium concentrations, a known β_2_-AR response, and increased serum glucose, insulin, and lactate. All three metabolic responses were blocked by nadolol but not bisoprolol, indicating that they occurred via β_2_-AR Interestingly, BRL 35,135 but not salbutamol increased serum FFA and glycerol concentrations (similar to findings in dogs [56]), but that also appeared to be a β_2_-AR effect. A recent study administered a high dose of mirabegron (100 mg q.d.) to 14 healthy women of various ethnicities for a period of 4 weeks [13]. Insulin responses in a glucose tolerance test were similar prior to and after 27 days of treatment. Taken together, these limited data do not support a relevant β_3_-AR-mediated insulin release in humans, which is similar to dogs but contrasts findings in rats and mice.

### 3.2. Cellular Glucose Uptake

Despite the controversial data concerning the role of β_3_-AR in mediating insulin release, several lines of evidence have indicated the possible involvement of β_3_-AR in enhancing glucose tolerance and uptake. El Hadri et al. attributed this phenomenon to the complex interaction between feeding/fasting status and the expression of β_3_-AR in AT [66]. Nevertheless, insulin stimulated glucose transport in rat adipocytes with a rank order implying a β_3_-AR involvement [67,68,69,70]. Several studies supported this claim. For instance, using CL 316,243 in transgenic mouse models with either β_3_-AR specific expression in AT or with β_3_-AR knockout mice, suggested that insulin release, food intake, and oxygen consumption induced by CL 316,243 is mediated by β_3_-AR in WAT [64]. Moreover, CL 316,243 seemed to increase glucose uptake in a tissue dependent manner, and variations were observed in different models of diabetes. CL 316,243 was effective in improving glucose uptake in BAT but not in inguinal WAT in T2DM mice. The opposite was observed in T1DM animals [71]. One study included different organs to examine the effect of BRL 37,344 in male Sprague Dawley rats. As expected, BRL 37,344 improved glucose uptake in skeletal muscle, heart, and diaphragm, in addition to BAT and WAT [72]. Other than that, a one week treatment with CL 316,243 (1mg/kg/day) induced glucose uptake in Wistar rat WAT but not in guinea pigs, alongside upregulation of GLUT4 mRNA expression in subcutaneous WAT (scWAT) and BAT of treated rats [73]. These data indicate a variation of β_3_-AR role and response in glucose uptake across species.

Functional evidence unravelling β_3_-AR induced glucose uptake in humans is limited; however, it is speculated that β_3_-AR agonists such as mirabegron improve glucose tolerance and uptake [74]. One study indicated that in obese subjects, mirabegron improved glucose tolerance and insulin sensitivity [75]. Another clinical trial indicated that a supratherapeutic dose of 200 mg of mirabegron in healthy subjects stimulated BAT glucose uptake and resting metabolic rate [76]. Other researchers proposed that glucose uptake in BAT both in human subjects and in vitro is modulated by GLUT4 and uncoupling protein 1 (UCP1) is diurnal bound [77]. On another note, cold induced BAT activation increased glucose uptake in the supraclavicular and paraspinal regions [78], but it remains unclear whether β_3_-AR are involved in this effect.

In conclusion, β_3_-AR agonists consistently seemed to improve glucose uptake in various organs in human and animal models. This effect was more prominent in BAT and was mostly associated with a better metabolic status. However, given the limited presence of BAT in adult humans, it remains unclear how much this contributes to systemic glucose handling. More functional studies must be conducted, especially in humans, to further support these conclusions.

## 4. Lipolysis and Adipose Tissue Remodeling 

AT is unlike other organs in the body. It has peculiar morphology, physiology, and function that differ based on the location and the shade of the fat pad [79]. WAT mainly consists of unilocular adipocytes with lower mitochondrial and UCP1 expression and a large single lipid droplet; it chiefly serves as energy reservoir in addition to secretory function of various adipokines and hormones [80,81,82]. Plus, it counts for almost 80% of total body fat that is primarily found in subcutaneous and visceral pools [83]. On the other hand, BAT is a more heterogenous pool of adipocytes; the majority are of multilocular morphology with higher mitochondrial and UCP1 levels and small lipid droplets, hence the brown color [79,84]. BAT is largely used in thermogenic responses, using UCP1 to dissipate energy as heat rather than ATP [85,86,87]. Thus, WAT and BAT store fat and can use such stores by lipolysis; however, WAT mainly serves as energy storage and BAT mostly to generate heat.

Accordingly, BAT plays an important physiological role in rodents and hibernating animals but less so in other groups of mammals, including humans. While it has long been assumed that adult humans lack BAT, more recent studies including those using fluorodeoxyglucose positron emission tomography have revealed the presence of at least some BAT in adult humans [88]. These were located differently than in rodents, i.e., mostly in the supraclavicular and neck regions. However, these new findings do not affect the more general concept that BAT plays only a minor role in adult humans as the prevalence of BAT does not exceed some tens of percent. This may be too little to mediate robust systemic lipolytic responses.

Human brown adipocytes have been described to be comparable to murine beige adipocytes rather than brown, which are distinguished by having intermediate characteristics of white and brown adipocytes (unilocular and multilocular, respectively) [89]. Beige AT expresses specific genes such as transcription factor *Tbx1*, fatty acid transporter *Slc27a1*, as well as CD40 and CD137 [89]. Beige (also known as brite) adipocytes are the latest to be identified among the different shades of adipocytes; they are multilocular with fewer mitochondrial and UCP1 count than brown, but with more lipid droplets than white adipocytes [89,90]. Hence, the term beiging or browning refers to the transformation of WAT into a pool with beige characteristics in response to wide range of stimuli, increasing its thermogenic capacity, which has been associated with numerous positive metabolic outcomes and a target to treat these anomalies [91]. 

Adrenergic activation is known to be a positive modulator of metabolism, adipose physiology, and activity in both human and animal models [76,92]. Still, divergence in adrenergic response and expression in AT have been documented across species [93,94]. Henceforth, in this section we will dwell further on the role of β_3_-AR and AT across species. 

### 4.1. β_3_-Adrenoceptor Expression in Adipose Tissue

Numerous reports have described the presence of β_3_-AR in rodent AT. This includes rat [24,67,95,96,97,98] and mouse WAT [99,100,101,102] and rat [24,96] and mouse BAT [42,99,100,101,102]. Among the two splice variants of murine β_3_-AR, the β_3b_-AR dominates in WAT, whereas the β_3a_-AR does so in BAT [100]. Of note, β_3_-AR mRNA expression in rodent AT markedly exceeds that of β_1_- and β_2_-AR [24]. β_3_-AR mRNA was also found abundantly in cell lines derived from murine WAT, e.g., 3T3-F422A cells [68,103,104]. The expression of β_3_-AR mRNA was markedly reduced in AT from ob/ob as compared to lean mice [102].

In contrast, the data on β_3_-AR expression at the mRNA level in human AT are not fully conclusive. Some investigators found it in human WAT [105,106,107], and in infant [106] and adult cervical and inter- and suprascapular BAT [92,108,109]; however, other reports did not confirm this [110,111]. Of note, most of these studies only reported qualitatively and lacked comparison to other subtypes. The technically most advanced study in the field, comparing expression of all three subtypes in more than 30 human tissues, reported that expression in AT was much lower than for the other two subtypes and below the detection limit (β_1_ 2.29, β_2_ 12.60, β_3_ 0.19 fragments per kilobase of transcript length per million of mapped reads) [112] (Table 1). Thus, β_3_-AR mRNA is abundantly detected in rat and mouse WAT and BAT, whereas its detection in human BAT is inconsistent. Accordingly, β_3_-AR are the most abundantly expressed β-AR subtype in rodent AT [24] but the least abundantly expressed subtype in human AT [112]. Interestingly, Riis-Vestergaard et al. suggested, based on using both CL 316,243 and mirabegron, that sympathetic activation of human BAT is mediated by β_1_-AR activation and not β_3_-AR [113].

### 4.2. Lipolysis and Thermogenesis

#### 4.2.1. Non-Primate Animals Studies

Lipolysis studies in rodents were instrumental in postulating that a third β-AR subtype must exist [22]. Thus, initial studies from the Zaagsma group and others found that the rank order of potency of various agonists and antagonists to stimulate and inhibit rat AT lipolytic responses, respectively, did not match those at the β_1_- and β_2_-AR [17,18,19,54,114,115,116,117,118,119]. However, studies specifically performed in WAT mainly found an involvement of β_1_-AR [119]. Studies published after the cloning of the human [23], rat [24], and mouse β_3_-AR [25] confirmed the involvement of β_3_-AR in lipolytic responses in rats [120,121,122,123,124,125,126,127,128]. Thus, lipolytic responses in rats have a strong β_3_-AR involvement, although some of these studies have detected a β_1_-AR contribution. Interestingly, the lipolytic response isoprenaline or CL 316,243 in rat AT in the absence of insulin did not depend on the presence of the enzyme adenosine deaminase, whereas the stimulation in the presence of insulin was markedly attenuated by presence of the enzyme, with even stronger inhibition by the enzyme in the combined presence of insulin and glucose [69].

The initial report on the cloning of the murine β_3_-AR described that the potency of various agonists to stimulate cAMP formation in transfected CHO cells correlated well with that to induce lipolysis in rat brown adipocytes [25]. Subsequent studies confirmed the involvement of β_3_-AR in lipolytic responses in mice [44,129]. Studies in dogs also found that lipolytic responses are largely mediated by β_3_-AR [130,131].

#### 4.2.2. Human and Primate Studies

The role of β_3_-AR in lipolysis in humans and primates has been investigated extensively. An early study compared WAT from rat, dog, marmoset (*Callithrix jacchus*), baboon (*Papio papio*), macacque (*Macaca fascicularis*), and humans [132]. Isoprenaline was similarly potent in all six species. The intrinsic activity (expressed as fraction of maximum isoprenaline response) was about 1 in all species for noradrenaline. It also was about 1 for the β_1_-AR agonist dobutamine in rat and dog, about 0.9 in marmoset, and about 0.7 for baboon, macaque, and human. In contrast, it was about 1 for BRL 37,344 in rat and dog, about 0.6 for marmoset, and about 0.1 or less in baboon, macaque, and human. Other β_3_-AR agonists including CGP 12,177, CL-316,243, D7114, and SR 58,611 also had low potency and/or low efficacy in the latter three species. Antagonist experiments also supported the view that the lipolytic effects in baboon, macaque and human primarily involved β_1_- and β_2_-AR. Studies in isolated subcutaneous adipocytes from rhesus monkey reported that the β_3_-AR agonist L-750,355 concentration-dependently stimulated lipolysis; while its potency at the cloned monkey β_3_-AR was 28 nM, the lipolytic response did not reach an identifiable maximum even at 10 µM and was ≤20% of the isoprenaline response in concentrations close to its EC_50_ at the cloned receptor [133].

Several studies have explored the role of β_3_-AR in the regulation of lipolysis in humans, and the early studies have been reviewed previously [134]. One of the first experiments found that the activity of stereoisomers of β-AR antagonists could not be explained by involvement of β_1_/β_2_-AR in rats, but no such contradiction was found in human AT [19]. Later work from the same group found that BRL 37,344 stimulated lipolysis in human AT with much lower potency than in rat AT [118,126]. BRL 37,344 caused lipolysis in omental and subcutaneous white adipocytes by stimulating β_2_-adrenoceptors; lipolytic effects of CGP 12,177 may occur at least partly by a receptor distinct from β_1_-and β_3_-adrenoceptors [105]. Additionally, alprenolol, an antagonist with low affinity for β_3_-adrenoceptors, blocked the lipolytic response to isoprenaline in human but not rodent subcutaneous WAT [124], indicating that the human response is not mediated by β_3_-ARs. Others reported that the lipolytic effect of noradrenaline in human and monkey AT involved β_1_- and/or β_2_-AR with no evidence for an involvement of β_3_-AR [132]. Others found that lipolysis responses to CGP 12,177 were antagonized by bupranolol but only poorly by β_1_- and β_2_-AR antagonists; moreover, CGP 12,177 promoted lipolysis more effectively in human omental than in subcutaneous AT [135]. Thermogenic responses to an infusion of isoprenaline were attenuated by atenolol, indicating that they occurred by β_1_-AR stimulation. Several β_3_-AR agonists were tested in a follow-up study in human omental adipocytes: While some of them had lipolytic effects (BRL 37,344, CGP 12,177, CL 316,243, SM 11044 with some being only partial agonists), others did not (ICI D7114, SR 58611A, ZD 2079) [136], indicating that a lipolytic response in human AT may be limited to certain compounds and is not a universal response to β_3_-AR stimulation. Others also found that lipolytic responses in human WAT were only inconsistently achieved with β_3_-AR agonists [137]. 

Excitingly, these findings were negated by Cero et al. using mirabegron on human derived brown adipocytes. Mirabegron stimulated lipolysis and thermogenesis, while silencing β_3_-AR in BAT blocked these processes [109]. Moreover, the aforementioned Trp64Arg mutation in β_3_-AR gene (see Section 2.1) reduces lipolysis in human WAT in response to L-755,507 [33]. Furthermore, a dose of 100 mg of mirabegron was enough to induce thermogenesis in supraclavicular skin in humans, without inciting off-target binding in comparison to higher doses [14]. These contradicting lines of evidence furtherd the debate concerning the involvement of β_3_-adrenoceptors in human lipolysis. 

On a different note, thermogenesis is a hallmark of BAT activity that is chiefly achieved by sympathetic activation and generally associated with positive metabolic outcomes [46,50,84,85,92,109,138]. In healthy adult subjects, paracervical and supraclavicular brown adipose tissues were biopsied; these pools had 1000 times higher UCP1 expression than the adjacent WAT, with higher glucose uptake after cold exposure [86]. Interestingly, caffeine intake (leading to various stimulant effects based on its antagonism of adenosine receptors) was suggested to ignite BAT thermogenesis in humans since it induces overall positive metabolic effect in obese and non-obese subjects, including thermogenesis, lipolysis, improved glucose tolerance, and insulin [59,139,140]. Consequently, it was implied that caffeine might modulate β_3_-AR activation in BAT [141]. Since thermogenesis can be achieved by cold induction and other safe compounds such as caffeine, the use of β_3_-AR agonists to activate this process might not be a feasible option.

#### 4.2.3. In Vitro Studies

Brown adipocytes sourced from adult cynomolgus monkeys were the focal point of a study that delved into the roles of β-AR in triggering lipolysis and thermogenesis. This investigation, employing agonists for the three β-AR subtypes (β_1_, β_2_, and β_3_), namely denopamine, procaterol, and CGP12177A, unveiled their shared responsibility in these metabolic processes [142]. Another study on immortalized brown adipocytes showed the additive role of β-AR subtypes in increasing cellular cAMP formation, evidenced by the activation of adenylyl cyclase by noradrenaline after these cells being incubated with CL 316,243. Hence, this activation was achieved by multiple β-AR subtypes, not only by β_3_-AR. Moreover, all β-AR agonists induced UCP1 expression, and the maximal rate was obtained by isoproterenol (100microM) [104]. Still, primary cell culture of mouse BAT showed that β_3_-AR activation using BRL 37,344 was the most effective one in inducing UCP1 synthesis [143]. Also, heat production from brown adipocytes of older rats (40 weeks old) was lower compared to their younger obese littermates (12 weeks). Furthermore, thermogenesis was found to be activated equally in both groups by β_3_-AR agonist BRL 37,344 compared to non-selective agonist isoproterenol which exerted a significant lower response in obese rats [144]. Furthermore, mirabegron stimulated UCP1 expression in mouse brown adipocytes and 3T3-L1 cells [145]. Indicating the role of β_3_-AR in promoting UCP1 mediated thermogenesis in rodent adipocytes. 

Most recently, immortalized human brown adipocytes were studied using not only subtype-selective agonists but also receptor knock-down experiments to determine the β-AR subtype involved in lipolysis and UCP1 expression [113]. Lipolysis and UCP1 expression were stimulated by isoprenaline and dobutamine but not by procaterol, CL 316,243, or mirabegron. Similarly, knock-down of β_1_-AR attenuated the isoprenaline-induced UCP1 expression. These data strongly support the idea that β_1_-AR and not β_3_-AR is largely responsible for the modulation of human BAT.

In conclusion, a major contribution of β_3_-AR to lipolytic responses has consistently been shown in rats, mice, and dogs. On the other hand, the role of β_3_-AR in humans has been reported only inconsistently and equivocally. Additionally, where detected, β_3_-AR often played a smaller role than β_1_- and/or β_2_-AR. Henceforth, studying β_3_-AR involvement in murine models can be considered irrelevant to human physiology. 

### 4.3. Adipose Tissue Remodeling 

AT is a dynamic endocrine organ that exhibits physiological changes in response to a range of stimuli, both positive and negative [146,147]. These alterations mainly involve variations in the adipokines profile, cell heterogeneity particularly within brown and beige pools, and thermogenic capacity that is mainly driven by UCP1. This process is referred to as adipose tissue remodeling [146,147,148,149,150].

Sympathetic activation is essential to maintain a healthy function and plasticity of AT, by inducing positive remodeling [109,151,152,153]. Cold has been used as a natural sympathetic activator of BAT [78,154,155]. Pharmacological interventions have been used to modulate and activate AT as well. For example, mirabegron was found to improve scWAT dysfunction and to induce positive adipose remodeling indicated by increasing UCP1 expression and lipolysis in obese and insulin resistant human subject [75].

Notwithstanding, it has been debated whether the upper hand in sympathetic activation of AT is mediated by β_3_-AR or not across species. As shown in a recent study by Blondin et al., adrenergic mediated thermogenesis and lipolysis of BAT is driven by different adrenergic receptors. In humans, it was found to be mediated through β_2_-AR activation rather than β_3_-AR; while in rodents, the opposite was documented. Not only the activation but rather the expression of ARs was different; human BAT was noted to have a higher expression of β_2_-AR as well [94].

As stated before, adipose tissue, particularly BAT, exhibits considerable disparities between humans and rodents, encompassing differences in localization, physiology, morphology, and function. Importantly, BAT relative to body mass is notably lower in humans compared to rodents [87,156]. Different β-ARs subtypes are active and predominant as well. Indeed, β_3_-AR is believed to be the key modulator of positive AT remodeling in rodents [80].

In fact, treatment with CL 316,243 for one week was found to induce AT remodeling in Wistar rats [73]. It also doubled the metabolic rate and raised body temperature, induced WAT browning and activation in wild type C57Bl/6 mice [157]. In fact, CL 316,243 infusion in Wistar rats fed on high fat diet was found to increase FFA uptake and lipolysis in BAT as well [158]. Additionally, mirabegron treatment (2 mg/kg of body weight) for 3 weeks seemed to lower body weight and adiposity in high fat diet fed mice. It also reduced brown adipocytes’ size in the interscapular pool while increasing UCP1 expression, increasing beiging in the inguinal depot, while improving insulin sensitivity and glucose tolerance compared to the vehicle treated mice [145]. Moreover, knockout of β_3_-AR exhibited impaired lipolysis in mice [156], upregulation of β_1_-AR mRNA in BAT and WAT, and increased adiposity [42]. These findings emphasize the role of β_3_-AR in rodent AT in inducing metabolic activity. However, these positive metabolic alterations in BAT were produced by caffeine intake in obese and non-obese rodents [140,159], giving off more convenient options in inducing positive adipose remodeling than β_3_-AR agonists. 

The wide gap in results concerning the role of β_3_-AR in AT remodeling across species and models makes translational research of β_3_-AR agonists in targeting AT dysfunction in rodents less predictive for humans. 

## 5. Obesity 

Being a metabolic sink for glucose and FFA, BAT activation has been considered a corner stone in improving metabolic health, since it is correlated to improved glucose and insulin sensitivity [9,10,11,12,160]. Studies with ephedrine [46,47,48] and other sympathomimetic agents including various β-AR agonists had reported increased thermogenesis and weight loss in the genetically obese ob/ob mice [49,50] or a prevention of high-fat diet-induced weight gain [161]. Therefore, it was suggested that targeting β_3_-AR in BAT pharmacologically has therapeutic potential for metabolic dysfunction and obesity [12,13,14,22,162].

### 5.1. Non-Primate Animal Studies

Conclusive evidence regarding the β_3_-AR/AT axis in developing obesity and metabolic derangements has been presented in rodents. For instance, in lean and obese diabetic Zucker diabetic fatty rats (a model of type 2 diabetes), CL 316,243 infusion revealed an anti-obesity and anti-diabetes effect by improving glucose tolerance, insulin sensitivity, thermogenesis, mitochondrial biogenesis, WAT, BAT, and skeletal muscle glucose uptake and reducing plasma FFA levels [163]. Administration of CL 316,243 in rats fed on a high fat diet, seemed to increase energy expenditure, UCP1 in BAT, and prevented WAT hyperplasia [164]. Moreover, treating the ob/ob mouse model of obesity with BRL 37,344 seemed to rescue from metabolic dysfunction, by improving systemic levels of glucose, FFA and insulin. Similarly, treatment with CL 316,243 prevented the body weight gain induced by a high-fat diet in AJ mice [161]. Indeed, β_3_-AR agonists reduced fat mass without affecting lean body mass in multiple studies in ob/ob mice [22]. Interestingly, chronic infusion of 1 mg/d of BRL 37,344 for 20 days increased UCP1 mRNA expression in BAT compared to acute intervention for 1 day [165]. BRL 35,135 also caused weight loss in ob/ob mice and in Zucker diabetic fatty rats.

UCP-DTA transgenic mice with toxigene-mediated ablation of BAT were significantly obese on week 12 of western diet feeding, which was not stimulated by hyperphagia compared to the wild type. They also presented deleterious metabolic anomalies such as insulin resistance, glucose intolerance, and hyperlipidemia. Moreover, these mice seemed to have lower rates of GLUT4 and β_3_-AR mRNA, and increased expression of tumor necrosis factor-α compared to the control littermates [166]. Similar findings were reported by Lowell et al. using the same transgenic mouse model in the absence of western diet. Moreover, using CL 316,243 exerted 50% lower thermogenic activity compared to the wild type [167]. Another model of β_3_-AR knockout mice, showed that after 8 weeks of high fat diet, the mice had glucose intolerance with hyperlipidemia, increased adiposity of WAT with inflammatory markers compared to the wild type [156].

### 5.2. Human and Primate Studies

In contrast to rodents, non-human primates, such as prepubertal baboons, did not seem to exhibit metabolic activity in WAT and BAT in response to different β_3_-AR agonists including SR 58,611A, BRL 37,344, CGP 12,177, and CL 316,243. Furthermore, β_3_-AR mRNA was not found to be abundant in these fat pads as well, which was also not correlated to UCP1 expression [168]. 

Similarly, the results from human studies regarding BAT β_3_-AR modulation of obesity remain inconclusive. On one hand, body mass index and body compositions were found to be strongly associated with lower BAT activity in morbidly obese individuals as well as cold induced thermogenesis [169]. A study on BAT^+^ and BAT^-^ men with similar age, body mass index, and adiposity showed that after prolonged cold exposure, insulin sensitivity, glucose regulation, and resting metabolic rate were all increased in BAT^+^ subjects only [170]. These data indicate a discrepancy in metabolic impact based on the presence of BAT in these subjects. Moreover, the presence of BAT per se does not ensure BAT activation, as it might be affected by environmental factors such as cold. For instance, a study evaluated BAT activity by integrated positron-emission tomography and computer tomography scanning with ^18^F-fluorodeoxyglucose in 14 overweight and obese compared to 10 lean men. BAT activity was found during cold exposure but not under thermoneutral conditions, and it was lower in obese than in lean subjects [154]. Of note, comparing cold induced thermogenesis with mirabegron treatment in lean and obese subjects from both sexes showed that upon 10 days of placing ice packs on one thigh for 30 min was enough to induce beiging in subcutaneous AT in both legs. Meanwhile, 10 weeks of daily 50 mg of mirabegron showed signs of beiging indicated by UCP1 expression from the same pool. In addition to that, no sex differences were found in beiging of scWAT in both arms of intervention [155]. Intriguingly, ephedrine did not seem to induce thermogenesis in intrascapular fat in healthy subjects, although it induced oxygen intake, flood flow to the area, and skin temperature, in addition to lipolysis. These results indicate the possible increased temperature due to increased blood flow induced by sympathetic activation rather than thermogenesis, since brown adipocytes were not detected from the biopsies [171]. 

Importantly, it was suggested that mutations of β_3_-AR gene, especially Trp64Arg mutation, were associated with obesity and metabolic dysfunction in humans [30,33,37,172,173,174]. Contradictory data were reported, where scientists found no association between of β_3_-AR gene mutation and obesity or adiposity [32,175,176]. As such, results from two cohorts, the Québec Family Study and the Swedish Obese Subjects, concluded that Trp64Arg mutation in β_3_-AR gene was not associated with cardiometabolic insults, obesity, adiposity, or body composition change over the period of 12 years in the former. Similar outcomes were recorded in the latter, as this mutation was not associated with any weight gain over time and no difference was found in this gene between the obese and non-obese subjects [175].

The current body of evidence concerning the involvement of β_3_-AR in obesity remains inconclusive, leaving uncertainty about whether mutations in β_3_-AR contribute to the pathogenesis of obesity or aid in its management by activating it. This ambiguity casts doubt on the efficacy and adequacy of targeting β_3_-AR in AT as a therapeutic approach for tackling obesity.

## 6. Clinical Development Programs

Based on the promising results in rodents and on some of the gene polymorphism studies, several pharmaceutical companies launched clinical development programs for their β_3_-AR agonists in obese and/or diabetic patients. These have been reviewed by Larson [177], who had played an active part in the program by Merck & Co.

The Beecham program on BRL 26,830 was apparently the first to enter clinical development and has been tested in at least four trials. A short-term infusion of BRL 26,830 in obese subjects increased insulin sensitivity [178]. Chronic administration studies, published in abstract form only, found an increase in placebo-adjusted energy expenditure across three studies [177]. However, the overall effects on body weight were unimpressive, and they were accompanied by tremor [179], a typical adverse effect from β_2_-AR stimulation [180]. Therefore, this program was discontinued, and another with the backup compound BRL 35,135 was initiated. Administration of single oral doses of BRL 35,135 or of salbutamol in the absence and presence of antagonists were studied in healthy volunteers [65]. Decreases of serum potassium and increases of glucose, insulin, and lactate were apparently mediated by β_2_-AR; an increase in FFA occurred with BRL 35,135 but not with salbutamol. While both agonists increased basal metabolic rate, this was a β_2_ response for salbutamol and possibly included a β_3_ component for BRL 35,135. The induction of a thermogenic response to BRL 35,135 in non-obese men was confirmed in another study [181], but this did not determine the β-AR subtype being involved. Treatment of obese subjects for 10 days improved insulin sensitivity but did not lower body weight [182]. No subsequent studies were reported, indicating that the program was discontinued because the overall effects on body weight were discouraging.

CL 316,243 has good selectivity for β_3_- relative to β_1_- and β_2_-AR but its efficacy is only about 60% of that of isoprenaline [183]. Despite several studies showing beneficial effects in rats and in immortalized human brown adipocytes [113], it improved insulin effects with only moderate effects on energy expenditure in an 8-week, placebo-controlled clinical trials in healthy lean men [184].

After the phenylethanolamine RO 16-8714 had shown beneficial effects in rodents [95,185], its infusion in humans increased energy expenditure but also heart rate [186,187]. No follow-up studies were reported. ICI D-7114 has been studied in a 14-day, double-blind, randomized trial in obese patients but the results on energy expenditure and body weight also did not support further investigation [188]. Similarly, TAK-677 [189] (0.1 and 0.5 mg b.i.d. for 29 days) resulted in a statistically significant but small increase in energy expenditure relative to placebo in obese patients but did not affect body weight or fasting levels of glucose, insulin or FFA [190].

L-796,568 has been administered in single doses of 250 and 1000 mg to healthy overweight men in a placebo-controlled, 3-way cross-over trial [191]. The 1000 mg dose increased energy expenditure by about 8%, which was accompanied by an increase in plasma glycerol and FFA. While heart rate and diastolic blood pressure remained unchanged, systolic blood pressure increased by about 12 mm Hg. It was also tested in a 28-day placebo-controlled study with daily doses of 375 mg in non-diabetic overweight men [192]. The two groups did not differ in energy expenditure at study end, and glucose tolerance was not altered either. No additional clinical studies were reported.

Mirabegron [193] and solabegron [62] have been tested in phase II studies in obese/T2DM patients but no clinical outcomes were disclosed by Astellas and GSK, respectively; neither compound advanced to phase III studies in an obesity or T2DM indication. However, based on clinical availability of mirabegron for the overactive bladder syndrome indication [194], academic investigators have performed additional clinical studies. A supra-therapeutic single dose of mirabegron (200 mg) increased BAT metabolic activity in healthy male subjects as assessed by fluorodeoxyglucose in positron emission tomography [76]. In a follow-up study with single doses of 50 and 200 mg mirabegron, the effect on BAT metabolic activity increased more than dose-proportionally by the greater dose [92].

Based on the accumulated clinical evidence, it has been concluded that β_3_-AR agonists yielded “a statistically significant elevation in total energy expenditure but this did not translate into a biologically meaningful negative energy balance”, i.e., a releavant loss of body weight [177].

## 7. Why Do Rodent and Primate Studies Differ?

**Figure 1:**
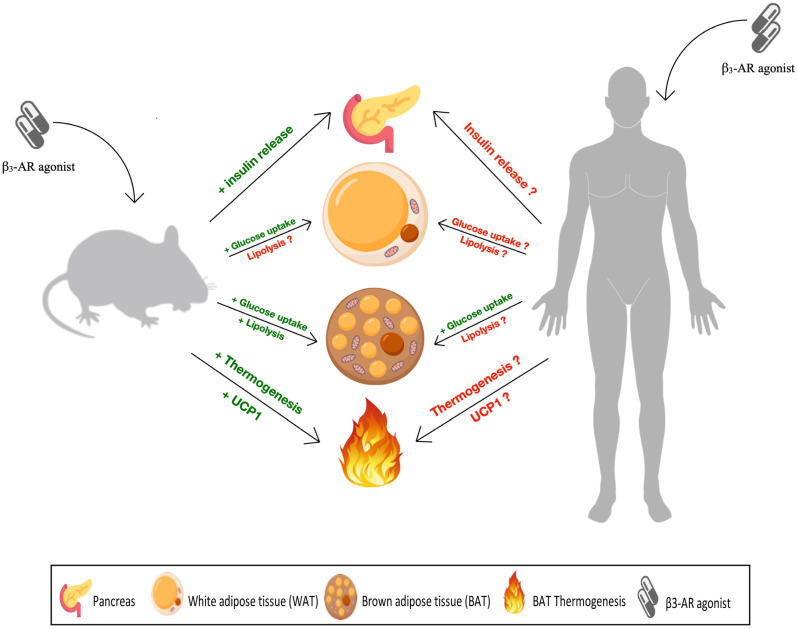
Differential metabolic impact of β_3_-adrenoceptor (AR) agonists across species. In rodents, β_3_-AR agonists, such as mirabegron, CL 316,243 and BRL 37,344, were found to promote positive metabolic implications by improving insulin release (indicated by the green plus sign), increasing glucose uptake in brown adipose tissue (BAT) and white adipose tissue (WAT) and lipolysis in the former only. In addition to upregulating uncoupling protein 1 (UCP1) expression and thermogenic capacity in BAT. On the other hand, data from human studies concerning insulin release, glucose uptake and lipolysis by WAT were inconsistent and inconclusive (indicated by the red question mark). Only glucose uptake human BAT was modulated consistently by β_3_-AR agonists. However, the data on β_3_-AR induced thermogenesis and UCP1 expression in human BAT were equivocal.

While the above data show that β_3_-ARs are a promising target for the treatment of obesity, and perhaps T2DM, in rodents, the data in humans and non-human primates are less conclusive and generally show quantitatively much smaller, if any, effect (Table 2, Figure 1). Accordingly, clinical development programs of β_3_-AR agonists by multiple pharmaceutical companies yielded small effects on thermogenesis that failed to translate into clinically relevant effects on body weights and led to the discontinuation of such programs. The above data indicate why a drug target promising in rodents was insufficiently responsive in humans.

First, the relative lack of efficacy in obese or diabetic humans is not due poor efficacy of drug candidates at the human receptor. Compounds that failed in clinical studies in obese and/or diabetic patients including mirabegron have been highly successful in other indications such as the overactive bladder syndrome. Second, a key factor in such species differences is the abundance of BAT, which is high in rodents but sparse in adult humans. It had already been concluded in the late 1990s that the body weight effects of β_3_-AR agonists (even in mice) depend on the presence of functional BAT [161]. While some maneuvers can promote the beiging of AT in humans, the presence of BAT or beige AT relative to body weight appears limited in humans. Third, β_3_-AR agonists promote pancreatic insulin release in rodents but have limited effects in humans. Fourth, the expression of β_3_-AR in AT appears to be considerably lower in humans than in rodents. While the lipolysis response in rodent WAT has a considerable β_3_-AR component, the response in human WAT is largely carried by β_1_-AR. Accordingly, the lipolytic and thermogenic responses in humans upon systemic administration are much smaller than in rodents. All of these factors in combination lead to a pronounced body weight reduction in rodents but not in men.

## 8. Conclusions and Future Perspectives

Rodent studies generally support the role of β_3_-AR as a target for the treatment of obesity and diabetes, whereas human studies mostly do not. This includes studies at the genetic level (gene knock-out studies in mice vs. genome-wide association studies in humans; see Section 2.3). Despite commercial efforts in various pharmaceutical companies (see Section 6 and Section 9), no β_3_-AR agonist has become a clinically available drug for the treatment of obesity and/or T2DM. To the contrary, at least nine β_3_-AR agonists have been tested clinically, some even in phase II studies, but failed to provide efficacy signals of sufficient strength to merit further development as anti-obesity/anti-diabetic drugs. In contrast, multiple β_3_-AR agonists have shown efficacy in patients with overactive bladder syndrome [62] and two of them, mirabegron [194] and vibegron [195], have become approved and guideline recommended treatments for this condition. In some cases, such as mirabegron, they had insufficient effects sizes in the obesity/T2DM indication but became approved drugs in the overactive bladder indication, indicating that the disappointing clinical data in the obesity/T2DM indication were not due to testing of an ineffective compound. Clinical development efforts in obesity and/or T2DM largely failed because of a sparse present of BAT in adult humans, differences in β_3_-AR expression, and other factors (see Section 6). As obesity is a market of huge potential commercial interest, it is telling that presently no major pharmaceutical company appears to be active in this space, including those who had active programs and discontinued them.

Some academic efforts have focused on inducing BAT or at least ‘beiging’ of WAT but it remains to be seen whether this will lead to a more successful use of β_3_-AR agonists as weight-lowering/anti-diabetic treatment. We personally are skeptical about these efforts for three main reasons: Firstly, clinical studies using β_3_-AR with various chemical structures have mostly found stimulation of thermogenesis in acute studies (single administration), whereas the limited chronic studies (multiple weeks) typically did not confirm this. This points to a possible role of desensitization of β_3_-AR [196]. Second, while β_3_-AR agonists generally are well tolerated, at least for mirabegron warnings on cardiovascular effects in a small fraction of patients have been issued [112]. It has been speculated that such adverse effects are related to a phenyl ethanolamine backbone, which is present in mirabegron and several other β_3_-AR agonists and may cause indirect sympathomimetic activity [197]. Moreover, at least mirabegron has additional off-target effects such as antagonism at α_1_-adrenoceptors [198,199,200]. Such off-target effects were neither reported nor are expected for the β_3_-AR agonists with other chemical backbones.

Third and most importantly, the key question is not whether β_3_-AR agonists will cause some weight loss relative to placebo; to make a clinical development program commercially viable, the candidate drugs must match the success of glucagon-like peptide 1 receptors such as semaglutide. Of note, compounds combining such agonism with that at receptors for glucose-dependent insulinotropic polypeptide, e.g., tirzepatide, apparently cause even greater weight loss [5]. We consider it highly unlikely that β_3_-AR agonists even with substantial beiging will ever reach such efficacy. This is further complicated that treatments with agents that first induce beiging and then stimulate β_3_-AR are highly complex, both in medical use and regarding a viable regulatory strategy. Not surprisingly, to the best of our knowledge, no major pharmaceutical company is maintaining clinical programs in this respect, including companies that have β_3_-AR agonists in their portfolio (see Section 9).

## 9. Patents

Following the lead of Beecham Pharmaceuticals (now a part of GSK) and its original drug discovery programs of β_3_-AR agonists as potential treatments of obesity [54], numerous pharmaceutical companies have filed patents seeking exclusivity for their β_3_-AR agonists as potential treatment of obesity and/or diabetes as reviewed elsewhere [201]. These patents cover thousands of compounds of various chemical structures [202]. They include patents from companies such as American Home Products, American Cyanamid Co., Asahi Kasei Pharma Corporation, Astellas Pharma, Bayer AG, Boehringer Ingelheim International, Bristol-Myers Squibb Co., Dainippon Pharmaceutical Co., Eli Lilly & Co., Fujisawa Pharmaceutical Co., Glaxo Group Ltd., Glenmark Pharmaceuticals Ltd., Imperial Chemical Industries Plc, Kaneka Corporation, Kissei Pharmaceutical, Merck & Co., Pfizer Inc., Sanofi SA, Smithkline Beecham Plc, Sumitomo Pharma, Tokyo Tanabe Co. Ltd., and Toyko Shinyaku Co. Ltd.; for details see [201]. Several of the compounds covered in those patents have entered clinical development (see Section 6), but none has proceeded beyond phase II or even been approved in the obesity/T2DM therapeutic field.

## Figures and Tables

**Table 1:** Quantification of β-AR subtype mRNA expression in human tissues. All data are shown as fragments per kilobase of transcript length per million mapped reads, a transcript abundance unit, in descending order of β_3_-AR expression and represent the median of samples from 3–7 patients.

	β_1_-AR	β_2_-AR	β_3_-AR
Ovary	0.02	0.79	6.89
Gall bladder	0.10	4.43	2.57
Placenta	28.77	5.99	2.53
Urinary bladder	0.29	6.63	1.54
Fallopian tube	0.09	4.38	0.64
Colon	0.75	2.07	0.54
Appendix	0.38	2.34	0.41
Prostate	4.01	9.64	0.29
Small intestine	1.18	1.66	0.25
Endometrium	0.07	2.20	0.22
Adipose tissue	2.29	12.60	0.19
Duodenum	0.73	1.54	0.19
Rectum	0.87	2.63	0.16
Brain	4.58	2.02	0.12
Myometrium	0.13	3.62	0.12
Stomach	0.76	6.68	0.11
Lung	6.55	18.01	0.07
Lymph nodes	0.11	3.08	0.07
Esophagus	0.99	8.55	0.03
Skin	0.16	5.60	0.02
Tonsil	0.31	5.51	0.02
Heart	11.57	4.92	0.02
Bone marrow	0.32	7.82	0
Spleen	0.90	6.90	0
Skeletal muscle	0.06	4.19	0
Liver	1.03	4.12	0
Salivary gland	4.93	2.54	0
Adrenal	0.16	1.48	0
Thyroid	0.21	1.07	0
Kidney	0.99	0.68	0
Pancreas	0.65	0.58	0
Testis	0.26	0.52	0

Adapted with permission from [112].

**Table 2:** Comparison of parameters linked to β_3_-AR and metabolism of rodent (rat, mouse) vs. human and other primate species. For details see main text.

	Rodent	Humans and Other Primates
BAT presence in adults	abundant	sparse
Insulin release by β_3_-AR agonists	+++	-
β_3_-AR expression in AT	+++	+
Glucose uptake in BAT	+++	+
Lipolysis/thermogenesis	+++	+
Lipolysis in WAT	β_3_-AR	β_1_-AR
Weight loss	++	inconclusive

## Data Availability

Not applicable.
